# Effects of *DGAT1* on milk performance in Sudanese Butana × Holstein crossbred cattle

**DOI:** 10.1007/s11250-022-03141-7

**Published:** 2022-03-25

**Authors:** Salma Elzaki, Paula Korkuć, Danny Arends, Monika Reissmann, Gudrun A. Brockmann

**Affiliations:** 1 Albrecht Daniel Thaer-Institute for Agricultural and Horticultural Sciences, Humboldt-Universität zu Berlin, Berlin, Germany; 2 Faculty of Animal Production, Department of Genetics and Animal Breeding, University of Khartoum, Khartoum, Sudan

**Keywords:** *Bos indicus*, Association analysis, Allele frequencies, Crossbreed

## Abstract

The improvement of milk production of indigenous Sudanese cattle such as *Bos indicus* Butana and its cross with Holstein is a major goal of the Sudanese government to ensure sufficient healthy nutrition in the country. In this study, we investigated the K232A polymorphism of *diacylglycerol acyltransferase* (*DGAT1*), a well-known modulator of milk production in other breeds. We determined allele frequencies and the allele effects on milk production. Therefore, 93 purebred Butana and 203 Butana × Holstein crossbred cattle were genotyped using competitive allele-specific PCR assays. Association analysis was performed using a linear mixed model in R. In purebred Butana cattle, the lysine *DGAT1* protein variant K232, which is found to be associated with higher fat and protein contents, as well as higher fat yield was highly frequent at 0.929, while its frequency in Butana × Holstein crossbred cattle was 0.394. Significant effects were found on milk yield (*P* = 7.6 × 10^−20^), fat yield (*P* = 2.2 × 10^−17^), protein yield (*P* = 2.0 × 10^−19^) and lactose yield (*P* = 4.0 × 10^−18^) in crossbred cattle. As expected, the protein variant K232 was disadvantageous since it was decreasing milk, protein, and lactose yields by 1.741 kg, 0.063 kg and 0.084 kg, respectively. No significant effects were found for milk fat, protein, and lactose contents. The high frequency of the lysine *DGAT1* protein variant K232 in Butana cattle could contribute to their high milk fat content in combination with low milk yield. In Butana × Holstein crossbred cattle, the *DGAT1* marker can be used for effective selection and thus genetic improvement of milk production.

**Supplementary Information:**

The online version contains supplementary material available at 10.1007/s11250-022-03141-7.

## Introduction

The improvement of milk production is a major goal of the Sudanese government to ensure sufficient and healthy nutrition and to meet the increasing demand for milk and dairy products in Sudan. One of the indigenous breeds in Sudan is the *Bos indicus* Zebu breed Butana which is known for its good milk yield and adaption to the extreme environmental conditions. To increase the productivity of Butana cattle, they are crossed with the high-yielding dairy breed Holstein–Friesian. Moreover, the productivity of Butana as well as Butana × Holstein cattle could be improved by developing sustainable management systems, breeding programs, and also genetic selection for higher milk yield.

The *DGAT1 (diacylglycerol acyltransferas*e) gene is located on bovine chromosome 14. The gene affects milk production traits in a wide-range of cattle breeds. Especially, the non-synonymous mutation K232A in exon 8 of *DGAT1* which causes an amino acid change from lysine to alanine has a strong effect on milk yield and composition (Grisart et al., 2002; Spelman et al., 2002; Winter et al., 2002). The lysine *DGAT1* protein variant K232 was found to be associated with higher fat and protein contents, as well as higher fat yield, but lower milk and protein yields (Bobbo et al., 2018; Bovenhuis et al., 2016; Li et al., 2021; Grisart et al., 2002; Spelman et al., 2002). Because of the strong negative relationship between lactose and fat content in milk across species (Fox, 2009), it is assumed that the lysine variant K232 also decreases lactose content. The frequency of the lysine variant K232 is high in *Bos indicus* cattle ranging from 0.80 to 1 (Abu Sara et al., 2015; Rahamattalla et al., 2015; Tantia et al., 2006; Kaupe et al., 2004; Spelman et al., 2002), but low in Holstein–Friesian cattle ranging from 0.21 to 0.63 (Grisart et al., 2002; Winter et al., 2002; Molee et al., 2012; Patel et al., 2009).

In this study, we investigated the K232A protein polymorphism of *DGAT1* using the DNA marker rs109234250 (14:611,019 G/A) in Sudanese purebred Butana cattle as well as Butana × Holstein crossbred cattle. We determined its allele frequencies and performed association tests with milk production traits to test the suitability of the *DGAT1* polymorphism as a marker for increasing milk yield and food security in Sudan.

## Material and methods

### DNA isolation and genotyping

Blood samples were collected from 93 purebred Butana cows (37 from Atbara Research Station and 56 from smallholders from different regions in North Sudan) and from 203 Butana × Holstein crossbred cows with varying levels of *Bos indicus* origin obtained from five farms in Khartoum North (44 Abbas Alreef Farm, 40 Judiciary Farm, 80 University of Khartoum, 19 Kuku Research Station, 20 University of Sudan). DNA was extracted from blood using a salting out procedure (Miller et al., 1988). The K232A polymorphism of *DGAT1* was investigated using the marker rs109234250 (14:611,019 G/A), whereby the allele A of this marker corresponds to the protein variant K232 with lysine. Genotypes were determined using allele-specific genotyping with competitive allele-specific PCR assays (KASP) (Kreuzer et al., 2013) (Supplementary Table 1). The genomic position is given with regard to the *Bos taurus* ARS_UCD1.2 genome assembly.

### Phenotypic data

One thousand one hundred twenty-seven test-day records for 203 Butana × Holstein crossbred cattle from five farms and 134 test-day records for 37 Butana cattle from Atbara Research Station were used in this study. Daily milk yield (MY) and milk composition were recorded monthly in 2017 and 2018. Samples of fresh milk were analysed using the milk analyser Lactoscan for fat (FC), protein (PC) and lactose (LC) contents in percentages. Fat yield (FY), protein yield (PY) and lactose yield (LY) in kilograms were calculated by multiplying the respective contents with MY. Based on the calving and sampling dates, the age at first calving, lactation number and days in milk were calculated. The total MY per lactation and the lactation length were recorded from the latest finished lactation of each cow. On average, 5.25 measurements per cow were recorded. Outliers were defined as values outside the mean ± 3 standard deviations and removed from the data set.

### Statistical analysis

Association analysis was performed only for Butana × Holstein crossbred cattle because the investigated *DGAT1* marker was not segregating among the 37 Butana cattle, which had phenotypic data available. Association analysis between phenotypic and genotypic data was performed with a linear mixed model using the lmer function implemented in R language for statistical computing (version 4.0.3). The model for testing the additive effect of the *DGAT1* marker was:

$$Y=X\beta +DGAT1+(1|animal)+error,$$

where *Y* is the test-day record of the respective trait, *X* is the matrix of significantly estimated fixed effects, *β* is the vector of the fixed-effects regression coefficients, *DGAT1* is the genotype at the *DGAT1* marker rs109234250 and *(1|animal)* is the animal as a random effect to compensate for repeated measurements of the animals followed by the error term. The fixed effects farm, lactation number, year of sampling, season of sampling, birth year, birth season, calving season, age at first calving in days and days in milk and were only included in the model, if they significantly contributed to the model tested using ANOVA (Supplementary Table 2).

## Results

### Overview of the investigated traits

The average MY per day was 2.4 times lower in Butana cattle (4.15 ± 1.40 kg) compared to Butana × Holstein cattle (10.12 ± 1.10 kg) (Table 1). Due to the low MY in Butana cattle, FY, PY and LY were also lower in Butana (0.25 kg, 0.22 kg and 0.21 kg, respectively) compared to Butana × Holstein crossbred cattle (0.43 kg, 0.36 kg and 0.49 kg, respectively) (Table 1). Correlations between FY, PY and LY with MY and between each other were high (*r* > 0.89) (﻿Table 2). In contrast, FC, PC and LC were higher in Butana (6.01%, 3.74% and 5.03%, respectively) than in Butana × Holstein crossbred cattle (4.30%, 3.59% and 4.84%, respectively). Lactation length of Butana cattle was shorter than in Butana × Holstein crossbred cattle (243 days vs. 309 days). Therefore, the total MY per lactation was lower in Butana compared to Butana × Holstein crossbred cattle (1222 kg vs. 3406 kg). Age at first calving was higher in Butana compared to Butana × Holstein crossbred cattle (1897 days vs. 998 days) (Table 1).

**Table 1:** Data overview for Butana and Butana × Holstein crossbred cattle per breed and farm. For phenotypes, average values including their standard deviations (SD) are shown

Breed	Unit	Butana	Butana × Holstein					
Farm		Atbara Research Station	Abbas Alreef Farm	Judiciary Farm	University of Khartoum	Kuku Research Station	University of Sudan	Average of all five farms
Number of animals	Counts	37	44	40	80	19	20	203
Number of records	Counts	134	271	218	423	83	132	1127
MY per day (SD)	kg	4.15 (1.40)	17.16 (1.59)	08.01 (1.20)	08.83 (1.43)	06.88 (2.70)	07.17 (2.23)	10.12 (1.10)
FY per day (SD)	kg	0.25 (0.08)	0.78 (0.09)	0.35 (0.06)	0.35 (0.07)	0.31 (0.14)	0.30 (0.08)	0.43 (0.20)
PY per day (SD)	kg	0.22 (0.05)	0.60 (0.06)	0.29 (0.04)	0.32 (0.05)	0.24 (0.09)	0.25 (0.08)	0.36 (0.10)
LY per day (SD)	kg	0.21 (0.07)	0.80 (0.09)	0.39 (0.06)	0.44 (0.08)	0.33 (0.13)	0.33 (0.10)	0.49 (0.20)
FC (SD)	%	6.01 (0.65)	4.53 (0.41)	4.42 (0.53)	3.97 (0.43)	4.74 (0.44)	4.30 (0.39)	4.30 (0.50)
PC (SD)	%	3.74 (0.11)	3.51 (0.13)	3.61 (0.13)	3.67 (0.14)	3.54 (0.13)	3.52 (0.14)	3.59 (0.20)
LC (SD)	%	5.03 (0.19)	4.65 (0.27)	4.92 (0.19)	4.99 (0.26)	4.78 (0.21)	4.56 (0.20)	4.84 (0.30)
Age at first calving (SD)	days	1896.60 (342.08)	793.47 (73.11)	1136.83 (147.92)	1036.83 (142.52)	1069.23 (94.76)	946.50 (103.68)	997.92 (171.41)
Lactation length (SD)	days	243.33 (63.50)	308.75 (5.05)	310.74 (9.83)	309.35 (11.86)	310.74 (6.06)	304.94 (8.59)	309.21 (9.66)
MY per lactation (SD)	kg	1222.88 (474.77)	5449.44 (603.64)	2629.46 (415.56)	3318.84 (570.89)	2276.08 (589.65)	2385.06 (593.91)	3406.01 (1218.03)
K232 of *DGAT1* (allele A at rs109234250)	Frequency	1.000/0.929*	0.386	0.475	0.400	0.658	0.575	0.394

^*^Allele frequencies for 37 Butana cattle for which traits were available and all 93 genotyped Butana cattle, respectively

**Table 2:** Pairwise correlations between investigated traits in Butana ×2 Holstein crossbred cattle. High pairwise Pearson correlations (*r* > 0.89) are highlighted in bold

Traits	MY	FY	PY	LY	FC	PC
FY	**0.930**					
PY	**0.987**	**0.913**				
LY	**0.974**	**0.892**	**0.985**			
FC	0.117	0.445	0.109	0.079		
PC	− 0.218	− 0.221	− 0.074	− 0.091	− 0.046	
LC	− 0.217	− 0.251	− 0.127	− 0.016	− 0.128	0.673

### Allele frequency of the K232 protein variant

When investigating the *DGAT1* marker rs109234250 (14:611,019 G/A), we observed that the frequency of allele A corresponding to the lysine *DGAT1* protein variant K232 was almost fixed in Butana. The allele was entirely fixed in the 37 Butana cattle from Atbara Research Station, but segregated at a high frequency of 0.929 for all 93 genotyped Butana cattle. In Butana × Holstein cattle, the average frequency of protein variant K232 was 0.394, whereas the frequency per farm ranged from 0.386 at Abbas Alreef Farm to 0.658 at Kuku Research Station (Table 1). The homozygous genotype of the lysine variant K232 was not observed at Abbas Alreef Farm, while the highest frequency of homozygous cattle with 0.368 was found at Kuku Research Station (Supplementary Table 3).

### Association analysis in Butana × Holstein crossbred cattle

The investigated *DGAT1* marker rs109234250 was not segregating in the 37 Butana cattle for which phenotypic data were available. Therefore, an association analysis with milk production traits could be performed for Butana × Holstein crossbred cattle only. We found significant associations with MY (*P* = 7.6 × 10^−20^, Supplementary Fig. 1), FY (*P* = 2.2 × 10^−17^), PY (*P* = 2.0 × 10^−19^), and LY (*P* = 4.0 × 10^−18^) (Table 3). The minor protein variant K232 which had a frequency of 0.394 was disadvantageous for the associated traits. It decreased MY by 1.741 kg, FY by 0.080 kg, PY by 0.063 kg and LY by 0.084 kg. No significant associations were found for FC (*P* = 0.370), PC (*P* = 0.812), and LC (*P* = 0.386) (Table 3).

**Table 3:** Associations between milk traits and *DGAT1* marker rs109234250 in Butana × Holstein crossbred cattle. The minor protein variant K232 of this marker had a frequency of 0.394. The β-estimates with regard to the minor allele (*β*_minor_), their standard error (SE (*β*_minor_)), and *p*-values from association tests are shown. *P*-values < 0.05 are highlighted in bold

Trait	*β*_minor_	SE(*β*_minor_)	*p*-value
MY	− 1.741	0.170	**7.6 × 10**^**−20**^
FY	− 0.080	0.009	**2.2 × 10**^**−17**^
PY	− 0.063	0.006	**2.0 × 10**^**−19**^
LY	− 0.084	0.009	**4.0 × 10**^**−18**^
FC	0.046	0.051	0.370
PC	0.004	0.015	0.812
LC	− 0.023	0.026	0.386

## Discussion

We found that the lysine *DGAT1* protein variant K232 is almost fixed in purebred Butana cattle. This is in line with studies in other *Bos indicus* breeds where the frequency of the lysine variant K232 ranged from 0.80 to 1 (Abu Sara et al., 2015; Rahamattalla et al., 2015; Spelman et al., 2002; Tantia et al., 2006; Kaupe et al., 2004). The high proportion of the lysine variant K232 might contribute to the higher FC and PC, and lower MY, FY and PY in Butana and other *Bos indicus* breeds compared to Holstein–Friesian cattle. The frequency of the lysine variant K232 in the investigated Butana × Holstein crossbred cattle is similar to studies in other Holstein–Friesian populations or their crosses where it ranged from 0.21 to 0.63 (Molee et al., 2012; Patel et al., 2009; Banos et al., 2008; Spelman et al., 2002; Thomsen et al., 2002). The differences in frequency of the lysine variant K232 among breeds might result from different breeding goals.

The association study provides evidence for *DGAT1* gene effects on milk production traits in the investigated Butana × Holstein crossbred cattle. The direction of effects of the lysine variant K232 on MY, PY, and LY was the same as in previous studies, decreasing them (Grisart et al., 2002, 2004; Spelman et al., 2002; Gautier et al., 2007; Schennink et al., 2007; Streit et al., 2011; Cardoso et al., 2011). We observed that the lysine variant K232 decreased FY, in contrast to what has been reported in literature. However, this might be due to the overall low MY in the crossbreed animals causing also a slight decrease in FY. Furthermore, the very high correlations between the associated traits in our study (*r* > 0.89) may justify the same direction of effects for MY, FY, PY and LY.

Different levels of Holstein genomic background in the different crossbred populations and differences in the management of the herds influence the MY of individual animals. We observed that cows at Abbas Alreef Farm produced around twice as much milk (on average 17.16 kg/day) as the cows on the other Butana × Holstein farms (Table 1). One reason for this are the excellent conditions with regard to farm management and feeding at Abbas Alreef Farm, which allows to make use of the genetic potential of Butana × Holstein crossbred cattle despite the harsh environmental conditions in Sudan. That is not to be taken for granted in Sudan considering the costs of feeding and management challenges at keeping high-yielding cattle. Thus, high variation of milk production traits and lactation properties between different farms is normal in Sudan. However, the average values reported in this study were in similar ranges compared to previously reported values for Butana × Holstein crossbred cattle (Supplementary Table 4) and Butana cattle (Supplementary Table 5). Another reason for the high MY is the higher amount of Holstein genomic background of crossbred cattle at the Abbas Alreef Farm compared to the other Butana × Holstein farms. This could be seen also in the allele frequency of the investigated *DGAT1* marker for the K232A polymorphism, where alanine variant 232A is the favourite variant in Holstein cattle: At Abbas Alreef Farm, where the Holstein genomic background is the highest, the frequency of the alanine variant 232A, which is beneficial for MY, is the highest. All in all, a negative trend between the frequency of lysine variant K232, and the daily MY in the five investigated farms is visible. It would be interesting to know to which extent the *DGAT1* marker could serve as a marker for the Holstein genomic background in crossbreeds with Holstein, where it is clear that this SNP was introduced from the Holstein breed. Furthermore, it would be desirable to consider the confounding factor of the Holstein genomic background. Unfortunately, neither pedigree data nor whole-genome data (e.g. from 50K SNP chip) are routinely collected in Sudan, so that we could not consider or investigate this factor in our current analyses. The only possibility to compensate for the farm-effect was to include “farm” as covariate in the association model. This farm-effect captured all differences between farms including farm management as well as Holstein genomic background.

Despite the small sample size in this study, we could confirm significant effects of *DGAT1* on Butana × Holstein crossbred cattle. With a higher sample size, it might be also possible to obtain significant associations for fat and protein contents in Butana × Holstein crossbred cattle.

In conclusion, the high observed frequency of the lysine *DGAT1* protein variant K232 in Butana cattle could explain their higher milk FC and lower MY. In Butana × Holstein crossbred cattle, the *DGAT1* marker can be used for selection and based on our results should lead to an increase in MY. Nonetheless, other factors such as the farm management and feed supply have to be improved in parallel to increase production efficiency.

## Supplementary Information

Below is the link to the electronic supplementary material.

Supplementary file1 (XLSX 205 KB)

## Data Availability

All phenotypic and genotypic input data used in this study can be found in Supplementary Table 6.
